# X-ray-induced reduction of Au ions in an aqueous solution in the presence of support materials and *in situ* time-resolved XANES measurements

**DOI:** 10.1107/S1600577514012703

**Published:** 2014-07-31

**Authors:** Yuji Ohkubo, Takashi Nakagawa, Satoshi Seino, Junichiro Kugai, Takao A. Yamamoto, Hiroaki Nitani, Yasuhiro Niwa

**Affiliations:** a Graduate School of Engineering, Osaka University, 2-1 Yamadaoka, Suita, Osaka 565-0871, Japan; b Institute of Materials Structure Science, High Energy Accelerator Research Organization (KEK), 1-1 Oho, Tsukuba, Ibaraki 305-0801, Japan

**Keywords:** synchrotron X-rays, radiation-induced reduction, X-ray absorption near-edge structure (XANES), Au-*L*_III_ edge

## Abstract

Synchrotron X-ray-induced reduction of Au ions in an aqueous solution with or without support materials is reported. To clarify the process of radiation-induced reduction of metal ions in aqueous solutions in the presence of carbon particles as support materials, *in situ* time-resolved XANES measurements of Au ions were performed under synchrotron X-ray irradiation. XANES spectra were obtained only when hydrophobic carbon particles were added to the precursor solution containing Au ions. Changes in the shape of the XANES spectra indicated a rapid reduction from ionic to metallic Au in the precursor solution owing to synchrotron X-ray irradiation. In addition, the effects of the wettability of the carbon particles on the deposited Au metallic spots were examined. The deposited Au metallic spots were different depending on the relationship of surface charges between metal precursors and support materials. Moreover, a Au film was obtained as a by-product only when hydrophilic carbon particles were added to the precursor solution containing the Au ions.

## Abstract 2

*In situ* time-resolved XANES measurements of Au ions in an aqueous solution in the presence of support materials were performed under synchrotron X-ray irradiation. The synchrotron X-ray-induced reduction of Au ions leads to the formation of Au nanoparticles on the carbon particles, acrylic cell or polyimide window. The deposited Au metallic spots were affected by the wettability of carbon particles.

## 1. Introduction   

There are many reports on the preparation methods for unsupported or supported metal nanoparticles in solution systems, for example the polyol method (Kurihara *et al.*, 1995 ▶; Patel *et al.*, 2005 ▶; Daimon & Kurobe, 2006 ▶), reverse micelle method (Liu *et al.*, 2002 ▶; Kinoshita *et al.*, 2003 ▶), ultrasonic irradiation method (Okitsu *et al.*, 1996 ▶; Mizukoshi *et al.*, 1997 ▶, 2007 ▶), hydrothermal synthesis method (Dawson, 1988 ▶; Adschiri, 2007 ▶) and impregnation method (Giorgio & Henry, 2002 ▶; Xu *et al.*, 2003 ▶). Our research group has prepared supported monometallic, bimetallic and trimetallic nanoparticles *via* irradiation with γ-rays and electron beams to aqueous solutions containing metal ions such as Au, Pt, Pd, Ag, Ru, Cu and Co, and support materials such as carbon particles, SiO_2_ particles, Al_2_O_3_ particles, Fe_3_O_4_ particles, TiO_2_ nanotubes, cotton fibers and nylon fibers. Supported metal nanoparticles have been studied and developed for various applications in the fields of medicine, biology and catalysis. There are various reports on the radiolytic preparation of supported metal nanoparticles and their applications (Seino *et al.*, 2008 ▶; Yamamoto *et al.*, 2010 ▶; Okada *et al.*, 2011 ▶; Kageyama *et al.*, 2012 ▶, 2013 ▶; Ohkubo *et al.*, 2013*a* ▶,*b* ▶). However, the process of radiation-induced reduction of metal ions in an aqueous solution in the presence of support materials is not clear. In particular, studies focusing on the behaviour of irradiated metal ions have not been reported, although in the field of radiation chemistry there are many reports that focus on radiation-induced active species such as the hydrated electron, hydrogen radical and hydroxyl radical (Belloni *et al.*, 1998 ▶, 2000 ▶; Belloni, 2006 ▶).

Table 1 ▶ shows a list of previously reports on X-ray-induced reduction and *in situ* measurements. In references 1–3 (Karadas *et al.*, 2005 ▶; Ozkaraoglu *et al.*, 2007 ▶; Fong *et al.*, 2011 ▶), metal salts were reduced *via* irradiation with normal X-rays using an X-ray photoelectron spectroscopy (XPS) device or soft X-rays using a synchrotron source while recording the XPS spectra; however, the metal salts were not in an aqueous solution during the measurements. In general, XPS measurement is performed under ultrahigh-vacuum conditions below 10^−5^ Pa and is therefore not suitable for solution samples. In contrast, the energy-dispersive X-ray absorption fine-structure (DXAFS) technique can be utilized under ordinary pressure and is therefore suitable for solid, solution and complex samples. In particular, the *in situ* time-resolved DXAFS technique has been recently applied to catalysts in solution for clarification of the reaction mechanism (Fiddy *et al.*, 2007 ▶; Tromp *et al.*, 2010 ▶; Asakura *et al.*, 2012 ▶). In references 4–8 (Jayanetti *et al.*, 2001 ▶; Mesu *et al.*, 2005 ▶, 2006 ▶; Mayanovic *et al.*, 2012 ▶; Ma *et al.*, 2013 ▶), metal ions were reduced in an aqueous solution *via* irradiation with synchrotron X-rays during DXAFS measurements. Considering further developments of the radiolytic preparation of metal nanoparticles, expected but unreported conditions for X-ray-induced reduction and *in situ* measurements are suggested in Table 1 ▶. In this study, we investigated the unreported conditions II (see Table 1 ▶) to clarify the process of radiation-induced reduction of monometallic ions in an aqueous solution in the presence of carbon particles as support materials. Au ions in an aqueous solution with or without carbon particles were irradiated with synchrotron X-rays for the reduction of the ions while recording X-ray absorption near-edge structure (XANES) spectra of the system.

## 2. Experimental   

### 2.1. Preparation of precursor solutions   

The materials used in the precursor solutions are given as follows. Ultrapure water (16 MΩ cm) produced by a Direct-Q system (Millipore) was deaerated by freezing with liquid nitrogen and was then used as the solvent. Tetrachloroauric acid tetrahydrate (HAuCl_4_·4H_2_O; Kanto Chemical) was used as the metal precursor. Untreated carbon black powder (Vulcan XC-72R, Cabot) was used as the hydrophobic carbon particles. Hydrophilic carbon particles were prepared by immersing the hydrophobic carbon particles in concentrated nitric acid (69 wt% HNO_3_; Wako) for 45 min at 363 ± 5 K while stirring. Carboxylic acid groups (COOH) would be formed on the surface of the carbon particles (Wang *et al.*, 2007 ▶; Osorio *et al.*, 2008 ▶). Aqueous solutions (1.6 mL) containing Au ions were prepared in 5 mL acrylic cells with 6 mm thickness, wherein a portion of the walls in the acrylic cells was cut away for the synchrotron X-ray path. Alternatively, a heat-stable polyimide (PI) film was used as the window material. An acrylic cell sandwiched between two silicon O-rings and two PI films was used as the solution cell.

### 2.2. *In situ* time-resolved XANES measurement while reducing Au ions   

**Figure 1:**
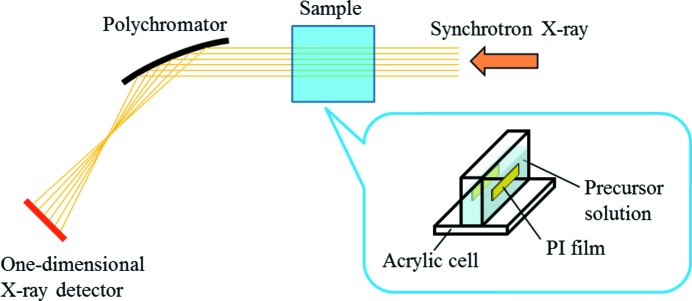
Schematic of the irradiation with synchrotron X-rays and *in situ* time-resolved XANES measurements of Au ions in the aqueous solution.

Fig. 1 ▶ shows a schematic of synchrotron X-ray irradiation and *in situ* time-resolved XANES measurements of Au ions in the aqueous solution. The acrylic cell containing the precursor solution was placed in the synchrotron X-ray path in front of the polychromator. The precursor solution was irradiated with synchrotron X-rays at the NW2A beamline of Photon Factory Advanced Ring for Pulse X-rays (PF-AR) at High Energy Accelerator Research Organization (KEK), Tsukuba, Japan. The precursor solution was continuously stirred during the irradiation. Chemical reductants such as hydrated electrons (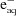) and hydrogen radicals (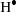) were formed by synchrotron X-ray-induced radiolysis of water (Muller *et al.*, 2004 ▶; Remita *et al.*, 2007 ▶), which resulted in the reduction of Au ions.

Changes in the chemical states of Au were investigated by comparing the time-resolved XANES spectra around the Au-*L*_III_ edge of the precursor solution with those of the reference materials HAuCl_4_ and a Au foil (Harada & Einaga, 2007 ▶; Evangelisti *et al.*, 2012 ▶; Ohkubo *et al.*, 2013*b* ▶; Ma *et al.*, 2013 ▶). *In situ* time-resolved XANES spectra around the Au-*L*_III_ edge (11850–12000  eV) were obtained using the DXAFS technique at the NW2A beamline while the precursor solution was irradiated with synchrotron X-rays, and consequently Au ions were reduced. To apply the DXAFS technique, the polychromator was controlled, and a one-dimensional X-ray detector was used. Each XANES spectrum was obtained for only 5 s. From the obtained XANES spectra, the ratio of ionic and metallic Au was calculated by linear-combination fitting.

Table 2 ▶ shows the sample IDs and experimental conditions of the precursor solutions and details of synchrotron X-ray irradiation. First, the deposited spots of metallic Au were investigated to determine the appropriate conditions of the precursor solution for obtaining analyzable XANES spectra. Second, the obtained XANES spectra were analyzed for tracking the process of synchrotron X-ray-induced reduction of Au ions in the aqueous solution.

## 3. Results and discussion   

**Figure 2:**
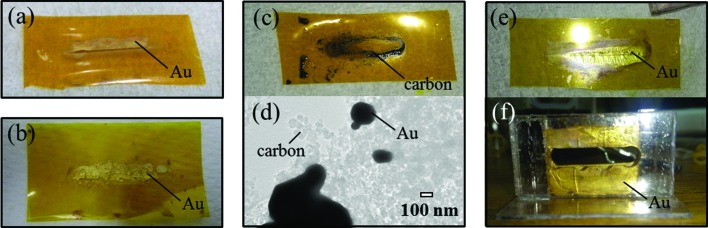
Photographs [(*a*)–(*c*), (*e*)–(*f*)] and TEM (*d*) image of metallic Au deposited *via* synchrotron X-ray irradiation: (*a*) PI film for sample 1, (*b*) PI film for sample 2, (*c*) PI film for sample 3, (*d*) carbon particles for sample 3, (*e*) PI film for sample 4, (*f*) acrylic cell for sample 4.

Table 3 ▶ describes the deposited spots of metallic Au for samples 1–4 and the analyzability of the *in situ* time-resolved XANES spectra. In all the samples the Au ions are reduced *via* synchrotron X-ray irradiation, but the deposited Au metallic spots are different. Fig. 2 ▶ shows photographs and a transmission electron microscope (TEM) image of metallic Au deposited *via* synchrotron X-ray irradiation. For sample 1, which has no carbon particles, metallic Au is observed on parts of the PI film (Fig. 2*a* ▶). Sample 2 has a similar behaviour as sample 1 (Fig. 2*b* ▶) although the undulator gap distances are different. These results for samples 1 (Fig. 2*a* ▶) and 2 (Fig. 2*b* ▶) indicate that metallic Au is deposited only on the spot irradiated by the synchrotron source in the absence of support particles. In contrast, for sample 3, which contains hydrophobic carbon particles in the precursor solution, no metallic Au is observed on the PI film although hydrophobic carbon particles are observed on the film (Fig. 2*c* ▶). Metallic Au deposit is observed on the carbon nanoparticles (Fig. 2*d* ▶) instead of on the PI film. On the other hand, for sample 4, which has hydrophilic carbon particles in the precursor solution, metallic Au is observed on the PI film (Fig. 2*e* ▶). These results for samples 3 (Fig. 2*c* ▶) and 4 (Fig. 2*e* ▶) indicate that the wettability of carbon particles as the support material affects the deposited spot. It is predicted that the hydrophilic carbon particles would repel the [AuCl_4_]^−^ anions because dissociated carboxylic acid groups (COO^−^) would be formed on the surface of the hydrophilic carbon particles, as previously reported (Wang *et al.*, 2007 ▶; Osorio *et al.*, 2008 ▶). Both the COO^−^ and [AuCl_4_]^−^ anions have minus charge, which would result in the generation of a repelling force between them. Besides, the deposition situation for sample 4 (Fig. 2*e* ▶) differs from those for samples 1 (Fig. 2*a* ▶) and 2 (Fig. 2*b* ▶). The amount of metallic Au deposited for sample 4 is obviously greater than those obtained for samples 1 and 2 despite a shorter irradiation time, and metallic Au for sample 4 is deposited like a plating film, as shown in Fig. 2(*e*) ▶. In addition, in the case of sample 4, metallic Au is deposited on not only the PI film (Fig. 2*e* ▶) but also the inner walls of the acrylic cell (Fig. 2*f* ▶). In fact, in the presence of hydrophilic carbon particles, metallic Au is deposited on not only the spots irradiated with synchrotron X-rays but also the non-irradiated spots. The results from Figs. 2(*a*)–2(*f*) indicate that (i) the synchrotron X-ray-induced reduction of the Au ions in an aqueous solution is not dependent on the wettability of support materials; (ii) metallic Au is easily deposited on hydrophobic surfaces; (iii) an increase in the solid–liquid interface area advances the reduction of Au ions.

For samples 1, 2 and 4, metallic Au was deposited on the PI film; therefore, it was difficult to analyze *in situ* time-resolved XANES spectra of these systems because the deposited metallic Au prevented the transmission of synchrotron X-rays, which resulted in inaccurate information regarding the changes in the chemical states of Au in the aqueous solution. In contrast, for sample 3, which had hydrophilic carbon particles, metallic Au was not deposited on the PI film; therefore, *in situ* time-resolved XANES spectra were successfully obtained during the reduction of Au ions *via* synchrotron X-ray irradiation.

**Figure 3:**
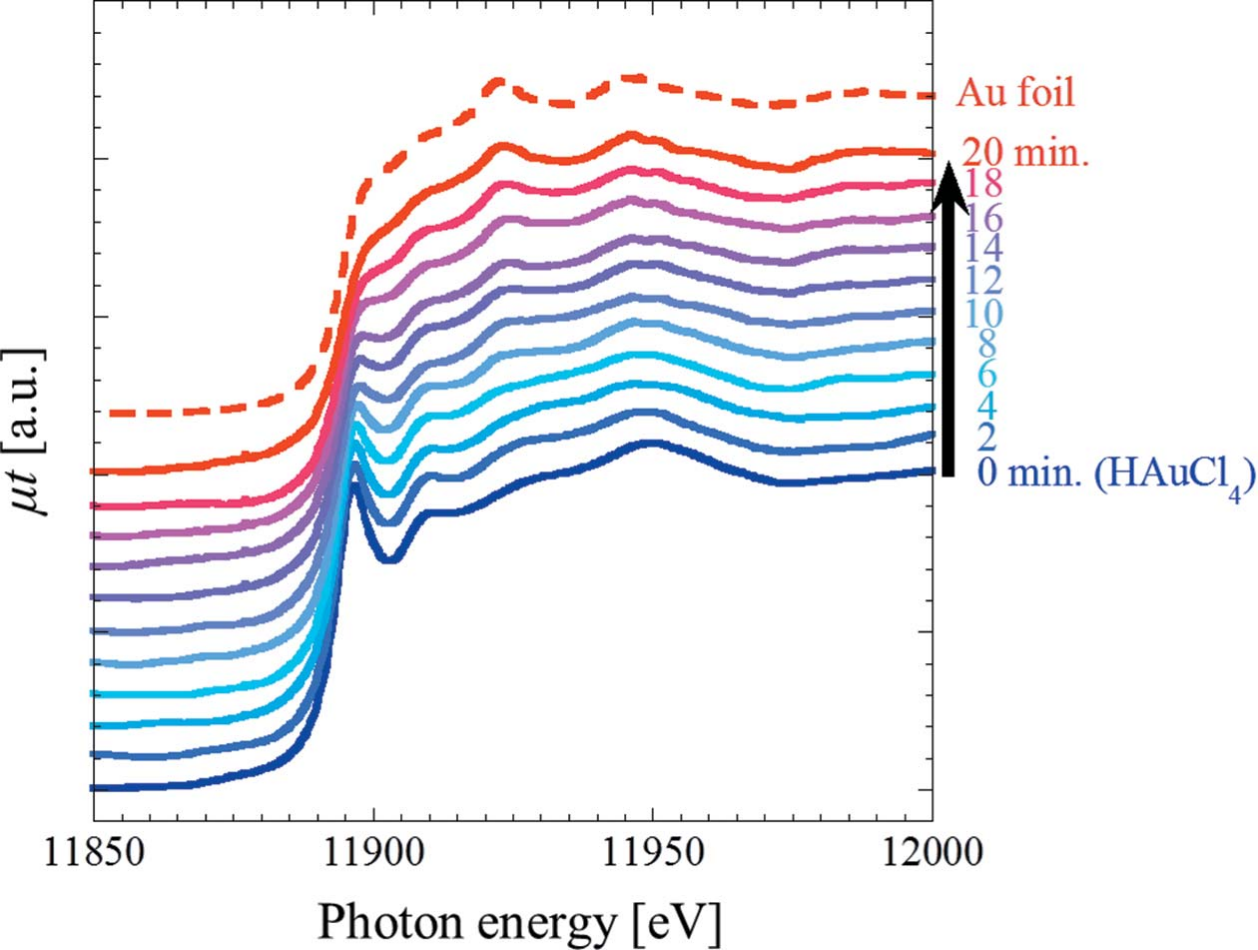
*In situ* time-resolved XANES spectra around the Au-*L*_III_ edge obtained for sample 3 during the synchrotron X-ray-induced reduction of Au ions in the aqueous solution.

**Figure 4:**
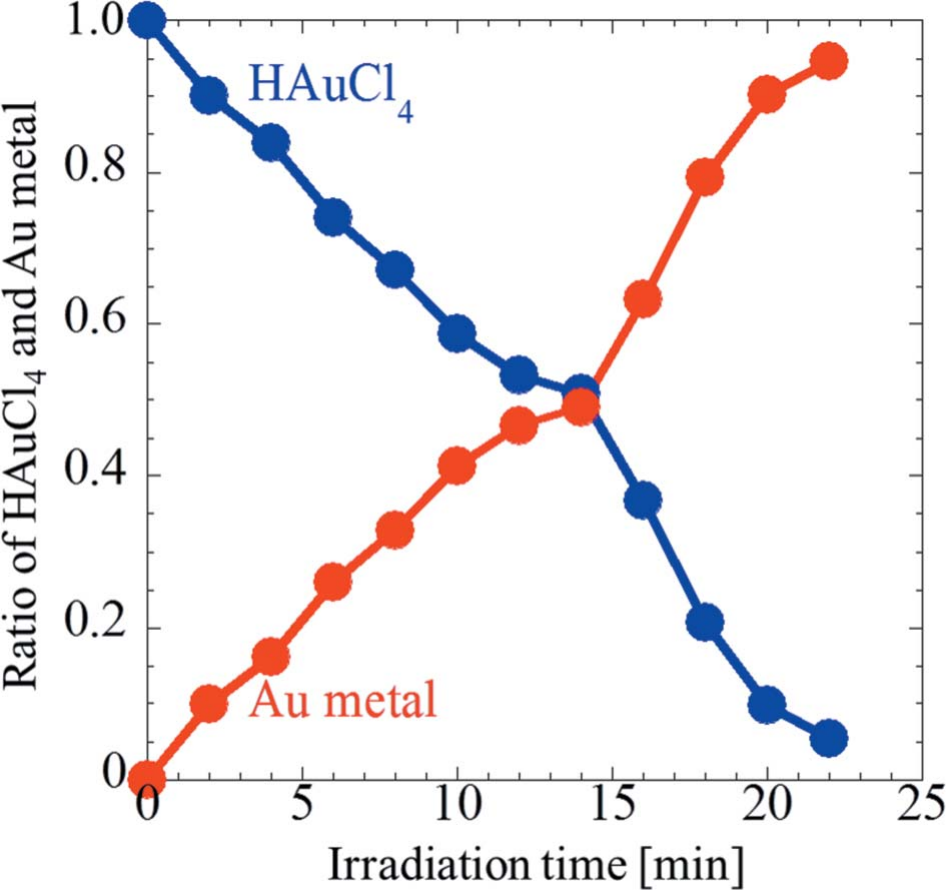
Ratios of HAuCl_4_ and metallic Au calculated using *in situ* time-resolved XANES spectra in Fig. 3 ▶ by linear-combination fitting.

Fig. 3 ▶ shows *in situ* time-resolved XANES spectra obtained around the Au-*L*_III_ edge for sample 3 during the reduction of Au ions in the aqueous solution *via* synchrotron X-ray irradiation. As the irradiation time is increased, the shape of the XANES spectra of sample 3 approaches that of a Au foil. Fig. 4 ▶ shows the ratios of HAuCl_4_ and metallic Au, calculated using the XANES spectra in Fig. 3 ▶ by linear-combination fitting. It is clearly observed that the ratio of Au ions decreases, whereas that of metallic Au increases as the irradiation time is increased. After irradiation for 22 min, the reduction of Au ions is almost complete. These results demonstrate that *in situ* time-resolved XANES measurements performed during synchrotron X-ray irradiation enable the tracking of radiation-induced reduction of Au ions in an aqueous solution.

## 4. Conclusion   

In this study, synchrotron X-rays were irradiated to precursor solutions containing Au ions with or without carbon particles. *In situ* time-resolved XANES measurements of the precursor solution were simultaneously performed. The following are the two main results from the measurements. First, the deposited Au metallic spots were dependent on the wettability of the carbon particles added to the precursor solution, which suggests that the wettability of carbon particles enables the control of the deposited Au metallic spot. In particular, the addition of hydrophilic carbon particles to the precursor solution leads to the formation of a Au film. This phenomenon would be useful as a novel technique to form metal films *via* irradiation with, for example, X-rays, γ-rays and electron beams. Second, the addition of hydrophobic carbon particles to the precursor solution enabled *in situ* time-resolved XANES measurements for tracking the reduction of Au ions in an aqueous solution. This behaviour could also be applied to study other metal ions. The relationship of surface charges between metal precursor and supports materials must be carefully considered. *In situ* time-resolved XANES spectra could be obtained for tracking the reduction of metal ions in an aqueous solution in the presence of support materials only if the appropriate materials are selected. Further studies on the synchrotron X-ray-induced reduction of bi­metallic ions in an aqueous solution and simultaneous monitoring by XANES spectroscopy are currently underway in our group and will be reported in a future publication (see Table 1 ▶, unreported conditions I and III).

## Figures and Tables

**Table 1:** List of previously reported research papers and unreported conditions on X-ray-induced reduction and *in situ* measurement

Paper ID	Year	Phase	Metal species	Support material	X-ray source	*In situ* measurement
Reference 1	2005	Solid	Monometal (Au)	No	XPS	XPS
Reference 2	2007	Solid	Bimetal (AuPt)	No	XPS	XPS
Reference 3	2011	Solid	Monometal (Au)	No	Synchrotron X-ray	XPS
Reference 4	2001	Liquid	Monometal (Cu)	No	Synchrotron X-ray	XAFS
Reference 5	2005	Liquid	Monometal (Cu)	No	Synchrotron X-ray	XAFS and UVVis
Reference 6	2006	Liquid	Monometal (Cu)	No	Synchrotron X-ray	XAFS and UVVis
Reference 7	2012	Liquid	Monometal (Fe)	No	Synchrotron X-ray	XAFS
Reference 8	2013	Liquid	Monometal (Au)	No	Synchrotron X-ray	XAFS
Unreported conditions I		Liquid	Bimetal	No	Synchrotron X-ray	XAFS
Unreported conditions II		Liquid	Monometal	Yes	Synchrotron X-ray	XAFS
Unreported conditions III		Liquid	Bimetal	Yes	Synchrotron X-ray	XAFS

**Table 2:** Sample IDs and experimental conditions of the precursor solution and irradiation with synchrotron X-rays

Sample ID	Input Au concentration [mM]	Solution volume [mL]	Dispersed support material	Gap distance [mm]	Irradiated beam width [mm]	Irradiation time [min]
Sample 1	80	1.6	No support	24	25	24
Sample 2	80	1.6	No support	32	25	20
Sample 3	80	1.6	Hydrophobic carbon	32	10	22
Sample 4	80	1.6	Hydrophilic carbon	32	10	10

**Table 3:** Deposited Au metallic spots for samples 14 and analyzability of time-resolved XANES spectra

Sample ID	On the PI film	On the acrylic cell	On the support material	XANES spectra
Sample 1	Yes	No	No support	Unanalyzable
Sample 2	Yes	No	No support	Unanalyzable
Sample 3	No	No	Yes	Analyzable
Sample 4	Yes	Yes	N/A	Unanalyzable
